# Impact of storage techniques on ovine temporomandibular joint discs composition and physicochemical properties

**DOI:** 10.3389/fbioe.2025.1725134

**Published:** 2025-12-03

**Authors:** Daniela Trindade, Cecília R. C. Calado, João C. Silva, Ana C. Maurício, Nuno Alves, Carla Moura

**Affiliations:** 1 Centre for Rapid and Sustainable Product Development (CDRSP), Polytechnic of Leiria, Marinha Grande, Portugal; 2 Veterinary Clinics Department, Abel Salazar Biomedical Sciences Institute (ICBAS), University of Porto (UP), Porto, Portugal; 3 Polytechnic University of Coimbra, Coimbra, Portugal; 4 Associate Laboratory for Advanced Production and Intelligent Systems (ARISE), Porto, Portugal; 5 ISEL—Instituto Superior de Engenharia de Lisboa, Instituto Politécnico de Lisboa, Lisboa, Portugal; 6 Institute for Bioengineering and Biosciences (iBB), The Associate Laboratory Institute for Health and Bioeconomy–i4HB, Instituto Superior Técnico (IST), Universidade de Lisboa (UL), Lisboa, Portugal; 7 Department of Bioengineering and iBB-Institute for Bioengineering and Biosciences, Instituto Superior Técnico, Universidade de Lisboa, Lisboa, Portugal; 8 Associate Laboratory i4HB - Institute for Health and Bioeconomy, Instituto Superior Técnico, Universidade de Lisboa, Lisboa, Portugal; 9 Animal Science Studies Centre (CECA), Agroenvironment, Technologies and Sciences Institute (ICETA), University of Porto, Porto, Portugal; 10 Associate Laboratory for Animal and Veterinary Science (AL4AnimalS), Lisbon, Portugal; 11 Research Centre for Natural Resources Environment and Society (CERNAS), Polytechnic Institute of Coimbra, Coimbra, Portugal

**Keywords:** temporomandibular joint disc, ovine model, biochemical composition, freezing time, storage, extracellular matrix

## Abstract

**Background:**

The temporomandibular joint disc plays a vital role in daily activities, and when it is compromised, it significantly impairs oral health and quality of life. The use of animal tissues for decellularized tissue engineering applications has been gaining interest, and an appropriate method for storing these tissues before processing has yet to be explored.

**Methods:**

This study characterizes the native temporomandibular ovine disc and compares storage protocols aimed at maintaining its morphology, biochemical content, and mechanical and thermal properties. Three storage protocols were tested: (i) freezing at −20 °C in phosphate-buffered saline (PBS) and thawing at 4 °C (PBS + 4 °C); (ii) freezing at −20 °C in PBS and thawing at room temperature (RT) (PBS + RT); and (iii) wrapping the discs in PBS-embedded gauze, freezing at −20 °C, and thawing at RT (Gauze + RT). Protocols were evaluated for short-term storage at 1, 7, and 14 days, and compared with a native and a collagenase-treated disc.

**Results:**

All conservation protocols induced changes, though less pronounced than the enzymatic degradation. The PBS + 4 °C and PBS + RT highlighted contrasting biochemical and mechanical outcomes, and thermal analysis revealed alterations to collagen structure. The Gauze + RT protocol preserved the biochemical content over time but exhibited a higher compression modulus on day 14.

**Conclusion:**

These results highlight how crucial it is to select adequate conservation techniques when preparing the TMJ disc for future studies.

## 1 Introduction

The temporomandibular joint (TMJ) comprises a fibrocartilaginous disc between the mandible condyle and the glenoid fossa-eminence articular complex of the temporal bone (Donahue et al., 2019), where the disc is crucial for the absorption of the loads. It presents notorious morphological variations and can be divided into the anterior, intermediate, and posterior regions (in the anteroposterior dimension). The intermediate region also presents variations, which can still be divided into medial, central, and lateral (in the mediolateral dimension). These differences are reflected in the content of cells, collagen type I, and glycosaminoglycans (GAGs), the major components of the TMJ disc (Acri et al., 2019; Trindade et al., 2021). The TMJ is highly predisposed to suffer from trauma or degenerative events that may lead to deviations or disorders in the condyle-disc complex, which are characterized as TMJ dysfunctions (TMDs) (Ivkovic and Racic, 2018). TMDs are present in 31% of adults and elderly and 11% of children and adolescents (Valesan et al., 2021) and highly impact the psychological and social experiences of patients (Taimeh et al., 2023). The dysfunctions that affect the disc include thinning, perforation, and displacement from the native position (Vapniarsky et al., 2018). Ultimately, these pathological changes can lead to more severe degenerative conditions, such as osteoarthritis and osteoarthrosis (Silva M. et al., 2020).

TMDs can be managed with medications, physiotherapy, and occlusal splints at the beginning of the disease. In a mid-stage, minimally invasive procedures are considered as intra-articular injections, arthrocentesis, and arthroscopy. Unfortunately, these methods lack the capacity to restore a damaged disc, and there is no consistently effective treatment or consensus on treatment choices (Dashnyam et al., 2018). For example, a meta-analysis showed that platelet-rich plasma (PRP) injections provide lower pain levels than hyaluronic acid (HA), and either of these injections is better after arthroscopy (Sakalys et al., 2020). Interestingly, PRP is as effective as arthrocentesis (Rajput et al., 2020), HA/corticosteroids, and arthrocentesis (Marzook et al., 2020). Discectomy, total disc removal, is largely used in more advanced cases of TMDs. However, despite helping to restore the mandibular movements and minimize the pain (Ângelo, Sanz, and Cardoso, 2022; Miloro et al., 2017), it does not prevent degenerative events, such as ankylosis (Wang et al., 2019).

Tissue engineering (TE) is a promising area for the development of novel therapies for disc dysfunctions. However, despite the similar incidence of knee and TMJ osteoarthritis, the latter lacks research, funding, and specialized doctors (Bielajew et al., 2021). One promising strategy might be the use of decellularized tissues since they can recreate a highly biomimetic microenvironment, recapitulating the main morphological, structural, and biochemical features of native TMJ tissue (Trindade et al., 2021; Trindade, Alves, and Moura, 2022). To this end, it is critical to define the animal model for the decellularization process. Different authors have investigated different animal models. Pig and minipig are suggested as the most suitable ones since they closely resemble the human anatomy and alimentary diet (Kalpakci et al., 2011; Lowe et al., 2018; Vapniarsky et al., 2017). However, access to the disc in these animal models is obstructed by the zygomatic arch, which covers the lateral aspect of the joint. In addition, such discs can be more difficult and expensive to obtain from a local abattoir (Almarza et al., 2018; Vapniarsky et al., 2017), preventing their standard use in pre-clinical research. On the other hand, the ovine model, like sheep and goats, is easy to obtain, inexpensive, and has an accessible surgical site (Almarza et al., 2018). In addition, compared to humans, they are similar in shape and structure, and different authors have already demonstrated their suitability for TMJ research (Ângelo et al., 2016; 2018; Labus et al., 2021; Bosanquet and Goss, 1987; Lee et al., 2022).

Another relevant point to be taken into consideration when biological materials are used is their preservation, as not only can it be difficult to obtain fresh tissue, but also sometimes it is not possible to test/use them immediately after extraction due to long experimental testing protocols. Thus, the study of freezing the TMJ discs for their conservation is still debatable; in the literature, only two studies address this subject. Allen & Athanasiou investigated from one to five freezing and thawing cycles at −20 °C during 6–18h followed by thawing at room temperature (RT), and observed no changes in the mechanical behavior of the hogs TMJ discs (Allen and Athanasiou, 2005). However, Calvo-Gallego et al. showed that freezing the discs for more than 30 days impacted their viscoelastic properties (Calvo-Gallego et al., 2017). Despite this, in both studies, only the intermediate/central zone of the disc was analyzed, and the animal model chosen was the pig. Similar results were found when freezing rat tendons, where one or two cycles of freezing at −20 °C or −80 °C appear to preserve biomechanical properties. However, for five cycles and long-term storage (∼9 months), tendon properties did deteriorate (Quirk et al., 2018). Conversely, only one cycle of freezing at −20 °C and thawing at RT produced minimal deterioration of biomechanical and electromechanical properties in bovine articular cartilage (Changoor et al., 2010).

In addition to preserving mechanical properties, freezing and thawing can also alter biochemical content, since collagen and GAGs are essential for maintaining tissue functionality. Moreover, as there is currently no standard protocol and, to the best of our knowledge, no comprehensive characterization on ovine TMJ discs, this work aimed to characterize this animal model and evaluate the impact of several conservation protocols for short-term storage on the disc’s native properties under routine laboratory conditions. Aiming at extracellular matrix (ECM) preservation, the analyses included disc morphology, biochemical composition, and thermal and mechanical properties. The impact of the conservation protocols on the properties of the disc was analyzed in comparison to a collagenase-treated disc. Overall, this study intends to serve as a base for future applications of this material in the TE field, especially focused on TMJ regeneration.

## 2 Materials and methods

### 2.1 Tissue preparation

TMJ discs were obtained from local butchers and dissected from lambs’ joints (7–15 months), in which the ligaments and retrodiscal tissues were carefully removed. All samples were morphologically evaluated in weight with a scale, in thickness with a digital caliper, and the mediolateral and anteroposterior dimensions were determined using the software ImageJ version 1.54d (NIH, Bethesda, MD, USA). Native discs were stored in 0.01 M phosphate-buffered saline (PBS) (pH = 7.4) at 4 °C until further use.

### 2.2 Storage conditions

After extraction, three storage conditions were tested based on the modification of previous studies on porcine TMJ discs (Calvo-Gallego et al., 2017; Allen and Athanasiou, 2005): *(i)* freezing the discs at −20 °C in PBS and thawing at 4 °C (PBS + 4 °C_X days), *(ii)* freezing the discs at −20 °C in PBS and thawing at RT (PBS + RT_X days) and *(iii)* wrapping the discs in a PBS embedded gauze and freezing at −20 °C followed by thawing at RT in PBS (Gauze + RT_X days). Moreover, different time intervals of freezing (X) were assessed for short-term storage: 1, 7, and 14 days, reflecting laboratory workflows in which TMJ discs are typically processed within days to a few weeks after collection. Discs were subjected to characterization immediately after thawing, which required ∼1h at RT and ∼3h at 4 °C for complete thawing.

### 2.3 Enzymatic digestion

Enzymatic digestion was performed using a collagenase type II solution (0.03% w/v; 125–250 U/mg, Sigma-Aldrich) at 37 °C for 9 h, adapted from (Fazaeli et al., 2016), with a reduced incubation time to account for the smaller size and thickness of ovine TMJ discs compared to porcine tissue(Almarza et al., 2018).

Native and collagenase-treated discs were lyophilized at 0.2 mbar for 24h in a freeze-drier (LyoQuest, Telstar, Japan) before further characterization. Afterwards, for the native disc, the five regions of the disc were separated. For the disc subjected to collagenase, once it had become a thin membrane, separating it into the five regions was impossible, so it was decided to cut out a random portion of the disc.

### 2.4 Water content

Water content for each disc’s region and the disc subjected to collagenase was calculated with the difference in weight obtained before and after lyophilization using the following equation:

$$\text{Water content }\left(\%\right)=\frac{\text{wet}\;\text{weight}-\text{dry}\;\text{weight}}{\text{wet}\;\text{weight}}\times 100$$

### 2.5 Sulfated glycosaminoglycans quantification

Sulfated GAGs quantification was performed in the native and the discs that underwent degradation, according to (Silva J. et al., 2020), using the 1,9-dimethylmethylene blue (DMMB) assay. Briefly, weighted lyophilized samples were digested in a 100 μg/mL papain (from papaya latex, Sigma-Aldrich) solution at 60 °C overnight, followed by combination with the DMMB solution (Sigma-Aldrich) for 5 min at RT in the dark. A standard curve prepared using chondroitin 6-sulfate (sodium salt from shark cartilage, Sigma-Aldrich) was used, and the absorbance was measured at 525 nm using a microplate reader (SPECTROstar Nano, BMG LabTech, Offenburg, Germany). Five samples (n = 5) were used for each experimental group and normalized to the DW of the discs.

### 2.6 Total and soluble collagen quantification

Collagen was also quantified for the native and the discs that underwent degradation. For soluble collagen quantification, weighted lyophilized samples were digested in a solution of 0.1% pepsin (from pig gastric mucosa, Roche) in 0.01 M hydrochloric acid (HCl) at RT for 48 h. Sirius red staining assay was used for the quantification as previously described (Keira et al., 2004). Samples were mixed with 50 µM Sirius Red (Direct red 80, Sigma-Aldrich) for 30 min at RT, followed by centrifugation at 10,000 rpm for 15 min. The pellet was dissolved in 0.5M NaOH, and the absorbance was read at 540 nm using a microplate reader (SPECTROstar Nano, BMG LabTech, Offenburg, Germany). A standard curve of collagen type I (rat tail collagen, Sigma-Aldrich) was used.

Total collagen was quantified with the hydroxyproline kit (Sigma-Aldrich), where weighted lyophilized samples were digested in 0.2 mL of 6 M HCL, and the concentration was determined by the reaction of oxidized hydroxyproline with 4-(dimethylamino)benzaldehyde. The conversion of hydroxyproline to collagen was performed by multiplying by 7.52 (Cross, Carpenter, and Smith, 1973).

Five samples (n = 5) were used for each experimental group, except for total collagen, where three samples (n = 3) were used. All data was normalized to the DW of the discs.

### 2.7 Fourier-Transform Infrared spectroscopic analysis

The molecular composition of the native discs, treated with collagenase and subjected to the conservation protocols, was also analyzed by Fourier-Transform Infrared (FTIR) spectroscopy using an attenuated total reflectance detection mode (Alpha FT-IR, Bruker) in the mid-infrared region (400–4000 cm^-1^), with a resolution of 4 cm^-1^ and 64 scans per spectrum. All spectra were corrected in relation to an analysis conducted without the biological sample. Spectra were acquired with OPUS® software (version 6.5, Bruker), and the five morphological regions of the disc were analysed (n = 3).

Pre-processing and processing were conducted on Matlab R2012b (Mathworks Natick, MA, USA). The second derivative spectra were based on a Savitzky-Golay filter with a second-order polynomial over a 15-point window, followed by uni-vector normalization. Specific bands of the normalized second derivative spectra were used to estimate collagen and GAGs, as previously shown to be correlated with these compounds (Rieppo et al., 2013; Rieppo et al., 2012b; Tint et al., 2019): *(i)* collagen by the negative band at 1338 cm^-1^, which is attributed to the CH_2_ side chains vibrations, *(ii)* sulphated GAGs by the negative band at 1052 cm^-1^, which is assigned to C-O stretching vibrations of the carbohydrate residues and SO_3_^−^ asymmetric stretching vibrations and *(iii)* sulphated and non-sulphated GAGs by the negative band at 1376 cm^-1^ that is related to CH_3_ symmetric bending vibrations. Since the negative peaks are analyzed in the second derivative, this implies that as the peak decreases, i.e., becomes more negative, the corresponding content of the target compound increases. These correlations were verified by comparison of the second derivative spectra and the non-derivative spectra. Therefore, to simplify writing, we will refer to whether the compound’s content increases or decreases wherever we refer to the bands.

Multivariate spectral analysis was performed by principal component analysis (PCA) and was conducted with the second derivative spectra between 800-1800 and 2800–3600 cm^-1^. Some spectrum regions were not considered to minimize noise amplification due to derivatives.

### 2.8 Thermogravimetric analysis (TGA)

The thermal properties of the lyophilized native discs, treated with collagenase and subjected to the conservation protocols, were studied using a Simultaneous Thermal Analyser, STA 6000 (PerkinElmer, Waltham, MA, USA) under nitrogen with a flow rate of 20 mL/min in the temperature range of 30 °C–500 °C and a heating rate of 10 °C/min (n = 3).

### 2.9 Mechanical testing under compressive stress

All samples were subjected to compression tests either immediately after extraction or immediately following collagenase treatment or thawing. All discs used for the study presented a thickness of 2.13 ± 0.33 mm, an anteroposterior dimension of 13.21 ± 1.15 mm, and a mediolateral dimension of 22.78 ± 1.24 mm. The disc area was calculated using ImageJ and obtained a value of 256.78 ± 45.55 mm^2^. A texture analyzer was used (TA.XTplusC, Stable Micro Systems, UK), and the assay parameters were: 1.2 mm min^-1^ extension rate, 490 N load cell, and the discs were compressed until a strain of at least 80%. The compression modulus was obtained from the elastic region of the graphs (n = 3).

### 2.10 Statistical analysis

Statistical differences between samples were evaluated on GraphPad Prism eight software. A repeated measurement two-way ANOVA with Sidak’s *post hoc* test was performed for morphological analysis. A two-way ANOVA with Tukey’s *post hoc* test was employed for FTIR spectroscopy analysis. For the remaining assays, such as TGA, biochemical quantifications, and mechanical tests, a one-way ANOVA was used with Tukey’s *post hoc* test. All tests were calculated with a confidence interval of 95%.

## 3 Results

### 3.1 Biochemical content evaluation

**FIGURE 1:**
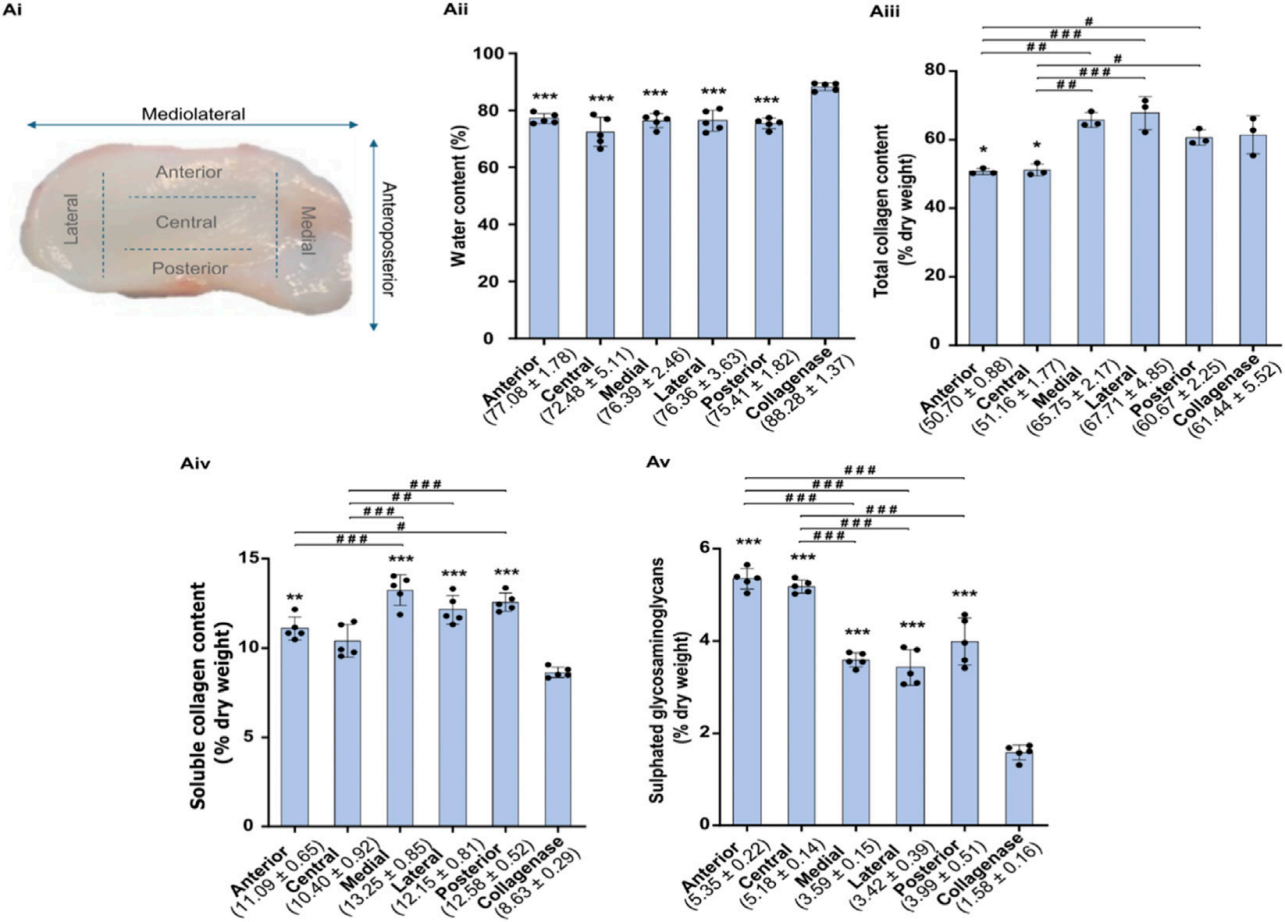
Ovine temporomandibular joint disc and its different regions: anterior, central, medial, lateral, and posterior **(Ai)**. Percentage of water **(Aii)**, total collagen **(Aiii)**, soluble collagen **(Aiv)**, and sulfated glycosaminoglycans **(Av)** content of the native and collagenase-treated discs. Significances are represented by *p < 0.05, **p < 0.01, and ***p < 0.001 when compared with the collagenase-treated disc, and represented by #p < 0.05, ##p < 0.01, and ###p < 0.001 when compared within the five regions of the disc.

The water, total and soluble collagen, and sulfated GAGs contents of the native discs were determined for the five regions of the disc (Figures 1–Ai). The extent to which disc degradation with collagenase affects water content and biochemical composition was also assessed. The differences in water content (Figures 1–Aii) between the five regions of the disc were not statistically significant, varying between 72.48% and 77.08%, with the lowest and highest values being attributed to the central and anterior regions, respectively. After degradation with collagenase, the water content increased to 88.28%, with all regions presenting statistically significant differences from the native disc. Total collagen content (Figure 1–Aiii) varied from 50.70% to 67.71% between regions, and soluble collagen (Figures 1–Aiv) varied from 11.09% to 13.25%, with the anterior and central regions having the lowest content for total and soluble collagen. The opposite was found for sulphated GAGs content (Figures 1–Av), varying from 3.59% to 5.35%, with the anterior and central regions having the highest content. When subjected to collagenase, on average, total collagen increased by 3.8%, soluble collagen decreased by 27.4%, and sulfated GAGs decreased by 63.2%.

**FIGURE 2:**
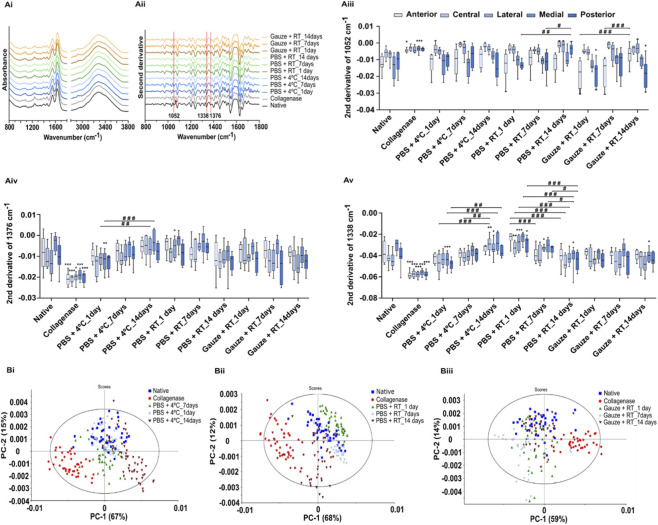
FTIR spectra **(Ai)**, second derivative spectra **(Aii)**, and box-plot graphs of the 1052 cm^-1^ **(Aiii)**, 1376 cm^-1^ **(Aiv)**, and 1338 cm^-1^ **(Av)** second derivative bands of the native, collagenase, and storage protocols: PBS+4 °C, PBS + RT and Gauze + RT. The arrow indicates the shift of the band. Principal components analysis of the second derivative spectra of the native, collagenase-treated discs **(Bi, Bii, Biii)** and conservation protocols: PBS+4 °C **(Bi)**, PBS + RT **(Bii)**, and Gauze + RT **(Biii)**. Statistical analysis was conducted using one-way ANOVA with Tukey’s *post hoc* test for figures A, and with two-way ANOVA with Tukey’s *post hoc* test for figures B. Significances are represented by *p < 0.05, **p < 0.01 and ***p < 0.001 when compared with the native disc, and represented by #p < 0.05, ##p < 0.01 and ###p < 0.001 are compared within the same conservation protocol.

FTIR spectroscopy showed that treatment with collagenase led to a shift in the band at 1052 cm^-1^ to 1058 cm-1 (Figures 2–Ai,Aii), resulting in an estimated decrease in sulfated GAGs in the anterior and medial regions (Figure 2–Aiii). As for sulfated and non-sulphated GAGs (indicated by the band at 1376 cm-^1^, (Figures 2–Aiv), and collagen (indicated by the band at 1338 cm-^1^, (Figures 2–Av), all regions of the disc estimated a significant increase after treatment with collagenase. As it will be explicated in the discussion section, this increase is in fact related to a degradation of these ECM components. For the preservation protocols, and compared to the native disc, the collagen content was the one that led to the most significant changes, specifically in the PBS + RT and PBS+4 °C protocols. These protocols also showed the most pronounced changes over time.

The PCA of FTIR spectra indicated different molecular compositions, with clear clustering among the native disc, collagenase-treated disc, and conservation protocols (Figures 2Bi–Biii). Day 7 showed the greatest similarity to the native group for the PBS + RT and PBS+4 °C protocols. However, the Gauze + RT protocol exhibited the least overlap on this day. The scores from the collagenase-treated disc are the ones further apart in space in relation to all other scores, pointing to a very different biochemical composition in relation to the native disc and in relation to any of the conservation protocols.

### 3.2 Morphological evaluation

**FIGURE 3:**
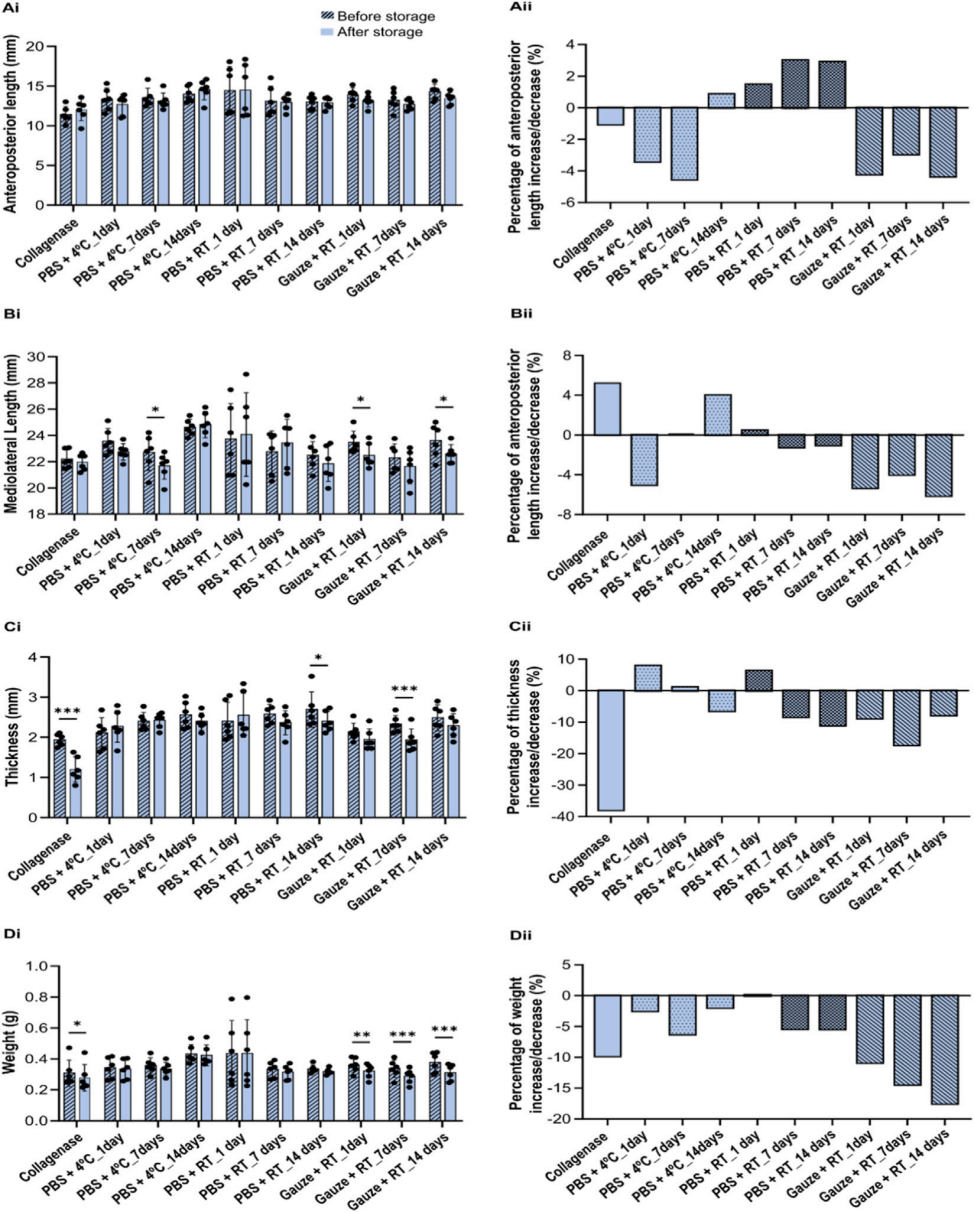
Relative anteroposterior **(Ai)** and mediolateral **(Bi)** dimensions, thickness **(Ci)**, and weight **(Di)** of the TMJ discs subjected to the collagenase and different conservation protocols: PBS+4 °C, PBS + RT, Gauze + RT, before and after the freezing storage and its corresponding percentage of increase or decrease **(Aii, Bii, Cii, and Dii)**. Statistical analysis was conducted with a two-way ANOVA with Sidak’s *post hoc* test, and differences are represented by *p < 0.05, **p < 0.01, and ***p < 0.001.

The native discs presented an anteroposterior dimension of 13.44 ± 0.65 mm, a mediolateral dimension of 23.34 ± 1.11 mm, a thickness of 1.62 ± 0.67 mm, and a weight of 0.39 ± 0.03 g. When subjected to the degradation and conservation protocols, no statistical differences were found for the anteroposterior length (Figures 3–Ai,Aii). For the mediolateral length (Figure 3–Bi.Bii), the protocols PBS+4 °C_7days, Gauze + RT_1, and 14 days led to a decrease of 4.5%, 4.2% and 4.4%, respectively. The thickness (Figure 3–Ci,Cii) was the most affected feature during the degradation process, with a decrease of 37.9%. The PBS + RT_14days and Gauze + RT_7days protocols also led to thinner discs with a decrease of 11.1% and 17.3%, respectively. Ultimately, for the weight (Figure 3–Di,Diii), the collagenase treatment led to a decrease of 9.8%. For the Gauze + RT protocol, the increase in the freezing time led to a higher percentage of weight loss: 10.9% after 1 day, 14.4% after 7 days and 17.5% after 14 days, in relation to the native disc.

### 3.3 Thermal analysis

**FIGURE 4:**
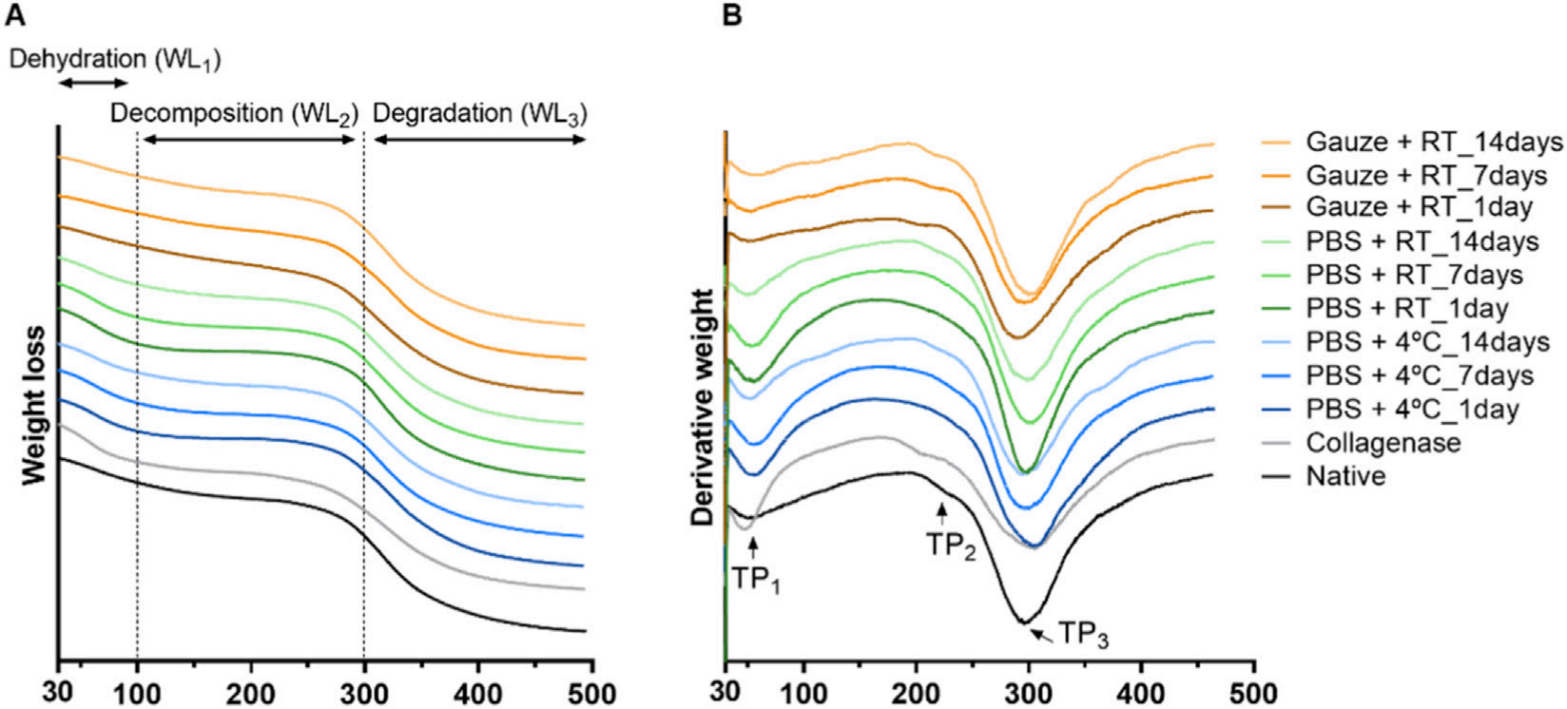
TGA **(A)** and DTGA **(B)** curves of the discs in the native form, collagenase-treated, and subjected to the different storage protocols.

The TGA and the derivative of the TGA (DTGA) revealed differences among the experimental groups. For analysis of the TGA results (Figures 4–A; Table 1), the dehydration phase (WL_1_: 30 °C < T < 100 °C), the decomposition phase (WL_2_: 100 °C < T < 300 °C), and the degradation phase (WL_3_: 300 °C < T < 500 °C) were considered. From the DTGA (Figures 4–B; Table 1), the denaturation peak of the hydrated collagen (TP_1_), the peak that corresponds to the conformational changes of the collagen molecule from a triple helix structure to the random coil (TP_2_), and the peak that corresponds to the bulk degradation of the collagen fibrils (TP3) were also evaluated (Rojas et al., 2023; Fertuzinhos et al., 2020).

**TABLE 1:** Mean and standard deviation values of the weight loss between 30 °C and 100 °C (WL_1_), 100 °C–300 °C (WL_2_), and 300 °C–500 °C (WL_3_), and the thermal peaks (TP_1_, TP_2_ and TP_3_) obtained from the TGA and DTGA analysis for the native, collagenase-treated, and subjected to storage discs. Statistically significant differences were calculated with one-way ANOVA with Tukey’s *post hoc* test, and differences represented by *p < 0.05, **p < 0.01, and ***p < 0.001 are compared with the native disc, and represented by #p < 0.05, ##p < 0.01, and ###p < 0.001 are compared within the same conservation protocol.

	WL_1_ (%)	WL_2_ (%)	WL_3_ (%)	TP_1_ (°C)	TP_2_ (°C)	TP_3_ (°C)
Native	11.60 ± 0.75	21.89 ± 0.97	41.55 ± 1.63	63.10 ± 0.88	237.37 ± 2.50	318.20 ± 0.83
Collagenase	15.90 ± 1.52*	20.07 ± 0.86	35.47 ± 0.46***	57.37 ± 1.81**	224.12 ± 1.50***	320.07 ± 1.26
PBS +4 °C_1 day	15.23 ± 1.21	16.03 ± 0.75***^,##^	40.66 ± 1.19	63.57 ± 0.22	-	320.71 ± 2.23
PBS +4 °C_7 days	15.69 ± 1.14	17.05 ± 1.19***^,#^	38.11 ± 0.63	64.75 ± 1.12	-	317.02 ± 1.48
PBS +4 °C_14 days	12.19 ± 0.65	20.26 ± 0.60^##,#^	38.91 ± 0.79	61.58 ± 0.66	233.98 ± 3.21	315.86 ± 0.41
PBS + RT_1 day	15.89 ± 0.34 *	16.19 ± 0.28***^,##^	39.56 ± 1.26	63.42 ± 1.34	-	313.31 ± 1.31^##^
PBS + RT_7 days	14.45 ± 1.03	17.24 ± 0.78**^,#^	40.71 ± 0.57	63.57 ± 1.84	-	317.83 ± 1.00
PBS + RT_14 days	12.50 ± 0.87	20.88 ± 1.23^##,#^	39.33 ± 1.29	60.61 ± 0.99	230.98 ± 1.63	321.92 ± 3.63^##^
Gauze + RT_1 day	8.83 ± 0.57	25.45 ± 2.20*^,#^	37.86 ± 2.47*	59.02 ± 2.24^#^	216.25 ± 4.46***^,##.###^	310.70 ± 2.98*
Gauze + RT_7 days	10.60 ± 3.30	22.85 ± 1.34	38.46 ± 1.31	60.95 ± 2.47	226.88 ± 0.68**^,##,#^	316.29 ± 1.92
Gauze + RT_14 days	11.80 ± 1.58	21.79 ± 0.58^#^	39.62 ± 0.91	63.85 ± 2.20^#^	234.36 ± 2.62^###,#^	314.52 ± 3.99

The native disc shows a weight loss of 11.60% in WL_1_, 21.89% in WL_2,_ and 41% in WL_3_. When degraded with collagenase, it releases a greater amount of water - WL_1_ (15.90%) and consequently reduces its weight loss during the degradation phase - WL_3_ (35.47%). For the conservation protocols, the samples were mainly affected in the decomposition phase - WL_2_. For the two protocols where the freezing is in PBS, the weight loss percentage is significantly lower on the first day, followed by a continuous increase until the seventh day, and then until the 14th day, where the values are similar to the native disc. Analyzing each protocol individually, this increase in weight loss shows that the 14th day is significantly different from days 1 and 7. The opposite is observed for Gauze + RT, in which on the first day of freezing the value is statistically significantly higher than the native disc, but with continued freezing, on days 7 and 14, the value is close to the ones observed for the native disc. This decrease in value also makes days 1 and 14 statistically different.

Regarding the thermal peaks observed in the DTGA, the native disc presented three occurrences: TP_1_ at 63.10 °C, TP_2_ at 237.37 °C, and TP_3_ at 317.20 °C. When the disc is degraded with collagenase, both the TP_1_ and TP_2_ appear at lower temperatures, being significantly different from the native disc. For the conservation protocols, the major differences are found in the TP_2_: the two protocols where the discs were frozen in PBS, during the first and seventh day, the peak is non-existent, but at the 14th of freezing, the value is similar to the native disc. As for the Gauze + RT protocol, the peak appears at lower temperatures on the first and seventh day. Moreover, in this protocol, the degradation peak - TP_3_ occurs at lower temperatures when the disc is frozen for 1 day. Analyzing this protocol individually, statistical differences are also found between the different freezing times for TP_1_ and TP_2_, in which increasing time leads to an increase in temperature.

### 3.4 Mechanical evaluation under compressive loading

**FIGURE 5:**
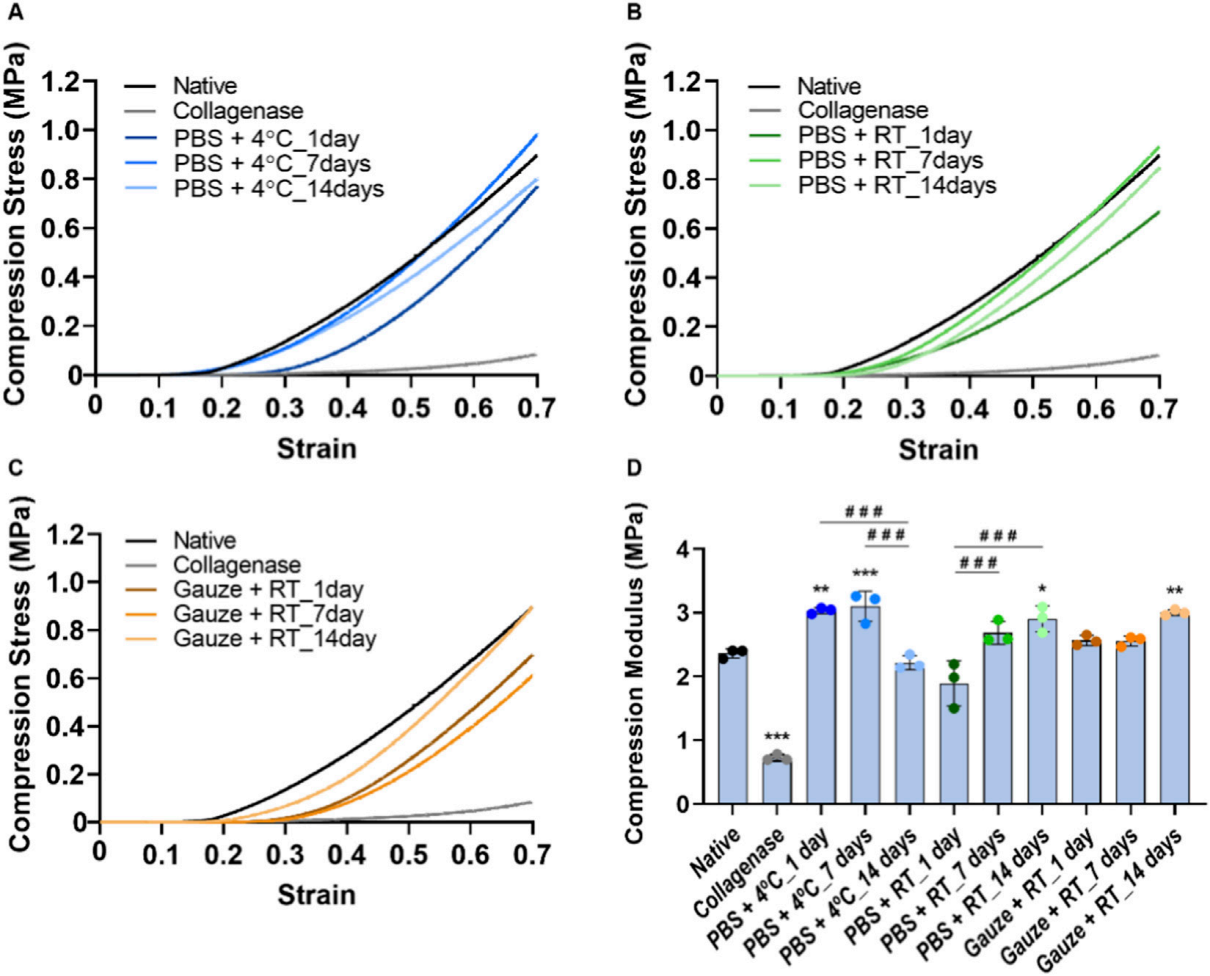
Stress-strain curves of the natives and collagenase-treated discs **(A–C)** and conservation protocols: PBS + 4 °C **(A)**, PBS + RT **(B)** and Gauze + RT **(C)**. Correspondent compressive modulus is represented in **(D)**. Statistically significant differences were calculated with one-way ANOVA with Tukey’s post-hoc test, and differences represented by *p < 0.05, **p < 0.01 and ***p < 0.001 are compared with the native disc, and represented by ###*p* < 0.001 are compared within the same conservation protocol.

Regarding the mechanical behavior evaluation, the native disc presents a compressive modulus of 2.36 ± 0.07 MPa, and after the collagenase treatment, the compressive modulus decreased 70% to 0.72 ± 0.04 MPa. For the PBS+4 °C protocol, during the first and seventh days of freezing, there was a 28% and 32% increase in the modulus, respectively, followed by a stabilization towards the native disc values at 14 days (Figures 5–A,D). The opposite was observed for the PBS + RT protocol, in which the modulus remained the same for the first and seventh days but increased by 23% after 14 days. For these two protocols, the difference between freezing for one or 7–14 days is also statistically significant (Figures 5–B,D). Finally, for the Gauze + RT protocol, the modulus increased by 27% after 14 days of freezing (Figures 5–C,D).

## 4 Discussion

The characterization of animal-derived tissues is extremely important to assess their potential to regenerate or replace human tissues. Different studies have evaluated different animal models and reported that the ovine model is suitable for TMJ research (Ângelo et al., 2016; 2018; Labus et al., 2021; Bosanquet and Goss, 1987; Lee et al., 2022). However, detailed information on its water and biochemical contents, particularly in terms of its soluble collagen, and subsequent comparison with a degraded disc has not yet been evaluated. Furthermore, an optimal conservation protocol is yet to be established so as not to alter the disc’s native properties and thus allow it to be used in TE strategies such as decellularization.

In the current work, it was observed that average values for native ovine TMJ disc were 75.5% ± 2.9% water, 59.2% ± 2.4% total collagen, 11.9% ± 0.7% soluble collagen, and 4.3% ± 0.3% sulfated GAGs by dry weight. These values are similar to the human TMJ discs (Kuo et al., 2010). Interestingly, it was also found that the anterior and central regions presented the lowest collagen content and the highest sulfated GAGs content for the ovine TMJ disc. The remaining regions display the inverse correlation. Interestingly, this inverse correlation between the amount of sulfated GAGs and type II collagen is usually found in the different zones of articular cartilage (Fu et al., 2020).

Collagenase treatment has a detrimental effect on the collagen network by cleaving the chains of the triple helix into small fragments and enabling the subsequent action of other enzymes, such as gelatinases (Powell et al., 2019; Billinghurst et al., 1997; Nimptsch et al., 2011; Almalla et al., 2023). These fragmentations have been observed in internal derangements of the human disc (Leonardi et al., 2007), so the use of this enzyme was important to assess the extent to which the conservation protocols damage the TMJ disc. The degraded native disc turned into a gel-like structure, which consequently led to a decrease in thickness and weight. In addition, as expected, the water content increased significantly since in osteoarthritic samples this also occurs due to the breakdown of the collagen network (Bank et al., 2000; Broom, Chen, and Hardy, 2001). The total and soluble collagen contents increased and decreased, respectively. Despite the increase in total collagen, this was only found for the anterior and central regions. This may be due to the portion of the disc analyzed, since, after freeze-drying, the disc remained in a paper-like structure, making it impossible to distinguish and divide the five regions. Another reason for the increase is due to the removal of sGAGs, which leaves collagen as a larger proportion of the tissue dry mass. So, in practice, collagenase treatment can lead to a stabilization or an increase in total collagen. This phenomenon is attributed to the limited efficacy of collagenases in cleaving insoluble collagen, which is characterized by a higher degree of crosslinked fibers (Eyre, 2001). Interestingly, it has also been reported that subjecting insoluble collagen type II to gastric pepsin results in an elevated degree of crosslinked aggregation within the collagen fibers, while for soluble collagen, the fibers almost disappear (Xu et al., 2021). It should be noted that collagenase treatment, by leading to the increase of total collagen, may negatively affect cellular behavior during tissue remodeling (Chung et al., 2004). Collagenase treatment also led to a significant decrease of sulfated GAGs, most probably due to the disintegration of the collagen network, resulting in GAGs release (Broom, Chen, and Hardy, 2001). This is concordant with Fazaeli et al. (Fazaeli et al., 2016) observation of a decrease in sulfated GAGs despite not finding a statistically significant reduction in collagen. The authors attributed this low reduction to the time and concentration of the enzyme. Compared to the present study, the animal model may also have contributed to this difference, as the ovine model presents a smaller disc when compared to the porcine model (Kalpakci et al., 2011), resulting in a much more effective action of the enzyme. Ultimately, all these changes in biochemical content led to a drastic reduction in the compressive modulus, which is in accordance with the reported for the porcine TMJ disc (Fazaeli et al., 2016; Fazaeli et al., 2019). These changes in the collagen structure after collagenase were further validated by TGA and DTGA analysis, where *(i)* a greater amount of water was released (WL_1_), *(ii)* the weight loss in the degradation zone (WL_3_) was significantly lower, and *(iii)* the peak corresponding to changes in the collagen triple helix (TP_2_) also appeared significantly earlier.

To evaluate the ECM of a cartilaginous tissue, usually diverse laborious and complex methods are conducted based on histology and biochemical testing (Tint et al., 2019). FTIR spectroscopic analysis may present complementary information concerning the sample molecular characterization while enabling a simple, rapid (a spectrum is typically acquired in 1 min) and economic (no expensive reagents are required) analysis (Baker et al., 2014; Araújo et al., 2020). The spectrum represents vibration modes of diverse functional groups and, consequently, may be used to evaluate the sample’s whole molecular composition and estimate the quantity of diverse biomolecules (Belbachir et al., 2009; Malek, Wood, and Bambery, 2014). Despite the advantages of the FTIR spectroscopy technique, it can present low specificity due to overlapping bands and even due to common bonds present in different molecules (Rieppo et al., 2012a). Spectra derivatives may enable the deconvolution of some overlapping bands, increasing the spectral resolution (Saarakkala et al., 2010). Indeed, diverse authors have used the FTIR technique to estimate collagen and GAGs in the articular cartilage of steers (Rieppo et al., 2012b), humans, and bovines (Rieppo et al., 2013).

Based on the analysis of the second derivative spectra, it was estimated that the collagenase treatment resulted in a decrease in the content of sulfated GAGs, with an average decrease of 62.9% for all regions when compared to the native disc, which is in accordance with the results obtained with the staining methods (63.2%). This decrease was also validated by the shift of the band to a higher wavelength (1052–1058 cm^-1^), as previously shown to occur when elastin content decreases (Cheheltani et al., 2014). For collagen, a 47.6% increase was inferred based on spectral analysis. A small part of this increase is in accordance with the 3.8% increase in total collagen as determined by the staining methods. However, most probably, based on the FTIR spectra, most of the increase results from the higher exposition of the aminoacids due to collagen fragmentation by collagenase. Thus, the increased infrared absorption of collagen is mainly due to a significant structural and conformation change of collagen and not due to an increased quantity of collagen. This is concordant with the observations based on polarized light microscopy of the loss of structural integrity after collagenase treatment (Fazaeli et al., 2016). There was also an increase in content for all GAGs (sulfated and non-sulfated). Although the band at 1376 cm^-1^ is generally used to quantify all GAGs, it also represents the absorption of glycoproteins (Rieppo et al., 2012b). Therefore, this absorption increase can also result from the significant increase in absorption of glycoproteins, due to the proteins' significant structural changes. Spectral PCA also reinforces this by pointing to a significant alteration of the molecular composition between the native and the collagenase-treated discs.

Regarding the conservation protocols, the one that most affected the morphology was the Gauze + RT, which led to a more dehydrated disc and, consequently, to its reduction in thickness and weight. Based on the ECM analysis by the second derivative, the disc’s composition was not significantly affected by the conservation protocols in comparison to native discs. However, some impact on the disc’s composition was observed in the spectra PCA, although it was much smaller than that observed with the collagenase treatment.

With TGA and DTGA, it was possible to observe the impact on the collagen structure. Both protocols in which the discs were frozen in PBS present similar results, where for days 1 and 7, the weight loss in the decomposition phase (WL_2_) was lower, and the decomposition peak (TP_2_) was non-existent. Gelatine, which is composed of denatured collagen, presents the collagen fibers in a random coil, meaning that the TP_2_ peak was also not found (Bozec and Marianne, 2011). Therefore, freezing and thawing caused this modification in collagen fibers. Contradictorily, for the Gauze + RT protocol, WL_2_ presented a significantly higher weight loss on day 1, and for TP_2,_ the peak for days 1 and 7 appears at a lower temperature due to lower thermal stability, as reported to occur in tendons (Giannini et al., 2008) and rat tail collagen-based hydrogels (Ozcelikkale and Han, 2016) upon freeze and thawing. Interestingly, day 14 showed a stabilization of the collagen network for all protocols by presenting results similar to the native disc. It is known that protein breakdown is caused by the formation of ice crystals (Ozcelikkale and Han, 2016), which ultimately appear to have a greater impact on the first and seventh day of freezing.

**FIGURE 6:**
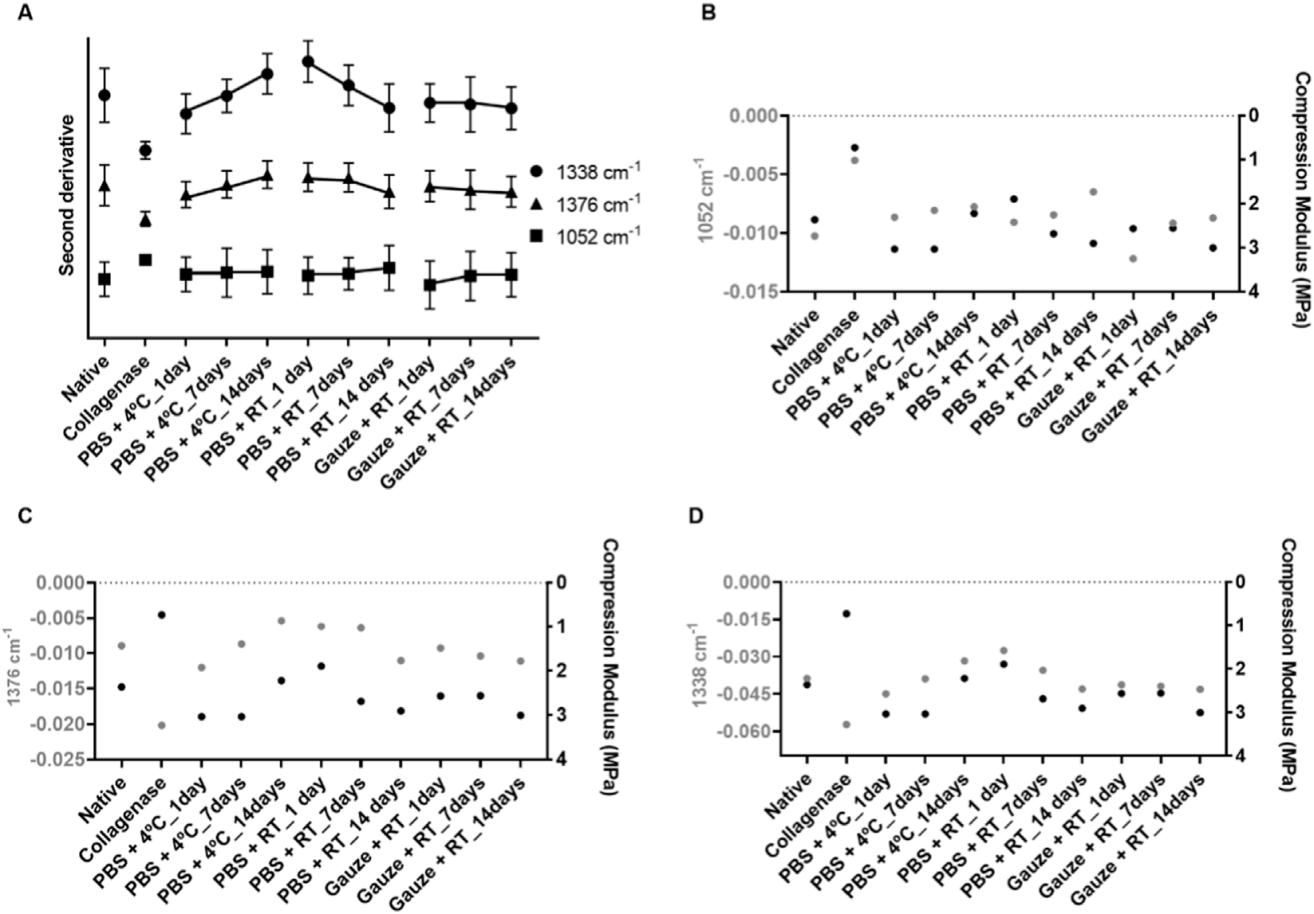
Bands of the second derivative spectra associated with sulfated GAGs (1052 cm^-1^), sulfated and non-sulfated GAGs (1376 cm^-1^), and for collagen (1338 cm^-1^) for all the protocols implemented: PBS+4 °C, PBS + RT, and Gauze + RT, regardless of the disc’s regions **(A)**. Relationship between compression modulus and sulfated GAGs **(B)**, sulfated and non-sulfated GAGs **(C)**, and collagen **(D)** for the native disc and the discs submitted to the different conservation protocols evaluated. Only the average of the results is presented.

It was also observed by FTIR spectroscopy, on all conservation protocols, a significant impact during the freezing period. To better analyze this, Figures 6–A shows the average values for all protocols, simultaneously considering all discs’ regions. It was possible to observe a pattern for sulfated and non-sulfated GAGs (1376 cm^-1^) and for collagen (1338 cm^-1^): for the PBS+4 °C protocol, increasing freezing times leads to a decrease of these compounds, whereas for the PBS + RT protocol, an increase of these compounds was found. Regarding sulfated GAGs (1052 cm^-1^), a decrease was observed for all conservation protocols as the freezing time increased.

Mechanically, the conservation protocols also had a detrimental effect on the compressive performance of the discs. PBS+4 °C led to a stiffer disc on the first and seventh of freezing, while PBS + RT was on the 14th day. For Gauze + RT, Allen & Athanasiou (Allen and Athanasiou, 2005) also investigated this protocol and reported that the discs can be frozen up to 5 times without altering their viscoelastic properties. However, with the same protocol but with NaCl instead of PBS, Calvo-Gallego et al. showed that the viscoelastic properties change after 30 days of freezing (Calvo-Gallego et al., 2017). Although in both studies only the intermediate/central zone was analyzed, and the animal model chosen was the pig, these results are in agreement with those found in the present study, as with this protocol, there were alterations in the compression capability after 14 days frozen. This alteration was defined to be due to the loss of interstitial fluid in the discs, since a more dehydrated disc was found.

The mechanical performance of the TMJ disc is defined by the cooperation of the different ECM components, and it is characterized as a viscoelastic structure, as it helps to absorb stress and distribute loads on the disc, cartilage, and bone components (David and Elavarasi, 2016; Fazaeli et al., 2019). In order to find out if there is a relationship between the quantitative results from the FTIR bands and the mechanical behavior, three graphs were plotted, in which only the average is presented for better visualization. Figures 6–B presents the relationship between sulfated GAGs (band 1052 cm-1) and the compression modulus. It was observed that both parameters for collagenase-treated and PBS + RT_1 decreased, while for the Gauze + RT_1days and Gauze + RT_14days, they increased. However, for part of the remaining protocols, the decrease of sulfated GAGs content seems to led to an increase in the stiffness of the disc. Regarding the sulfated and non-sulfated GAGs content (Figures 6–C), it was observed that with the increase in the biochemical content, there is also an increase in the compression modulus. The same is found in the relationship with the collagen content (Figures 6–D). The protocols that such a pattern is not extrapolated are the PBS+4 °C_7days for sulfated GAGs and PBS + RT_7days for sulfated GAGs and collagen. Regarding the discs treated with collagenase, this pattern is also not found because, as explained above, the increase in biochemical content for collagenase-treated samples in the FTIR analysis is actually indicative of its fragmentation. Furthermore, through all the characterizations reported in this study, no profile similar to collagenase-treated samples was found in the discs subjected to the protocols.

Although it has been reported that the collagen content is related to the tensile capacity of the TMJ discs, Detamore et al. highlighted the challenge of establishing a correlation between the content of sulfated GAGs and the compressive performance (Detamore and Athanasiou, 2003). Furthermore, Willard et al. pointed out that even after removing 96% of the sulfated GAGs using chondroitinase, there was no significant change in the instantaneous compressive modulus (Willard et al., 2012). On the other hand, collagen appears to exert a substantial influence on the compressive capacity of the TMJ discs (Fazaeli et al., 2016; Park et al., 2008; Gutman et al., 2018). These results agree with those found in the present study, in which a stronger relationship between compression tests and collagen content was found when compared to sulfated GAG. For total GAGs, there also seems to be a high association. However, as referred above, this band seems to have influence from glycoproteins, so extrapolating that, the mechanical behavior is related to total GAGs is more complex.

In summary, for the discs frozen in PBS, it was possible to conclude that the type of thawing can result in opposite behaviors, as the results of the compression tests align with those obtained for the quantification of the biochemical content. However, regarding the morphological and thermal properties, the impact of the thawing method does not appear significant, as similar results were obtained. Conversely, when the thawing process is the same, different results are found across all evaluated parameters, demonstrating once again the influence of the freezing method on the disc’s native characteristics. Our results suggest that all the protocols induced alterations in the native properties of the ovine disc, so in the case of using the full disc for TE strategies, these should be taken into account. However, if future studies involve using the disc in powder form, we recommend storing the discs in Gauze + RT for 14 days, as the dehydration and mechanical property changes observed by the 14th day become negligible.

The results of this study have significant translational implications for TE of the TMJ disc. We provide comprehensive guidelines for the design of biomimetic constructs by characterizing the native ovine fibrocartilaginous disc in terms of morphology, biochemical composition, thermal behavior, and mechanical properties. Furthermore, the evaluation of different conservation protocols offers practical insight into how native properties can be preserved, should this tissue be required for future experimental work. However, several limitations must be acknowledged. Only a subset of conservation conditions and periods was investigated, as long-term storage, lower temperatures, or controlled-rate freezing may yield different results. Second, the tests did not address how conservation could influence subsequent processing steps, such as decellularization efficiency or matrix stability, which are also important for future applications. Third, the relatively small sample size (n = three to five per condition) can reduce the statistical strength of the analysis. Despite these limitations, the present study provides a basis for standardizing tissue conservation and characterizing the ovine TMJ disc to support future translational TE studies.

## Data Availability

The original contributions presented in the study are included in the article/supplementary material, further inquiries can be directed to the corresponding authors.
